# Mechanisms of Myocardial Ischemia in Cancer Patients: A State-of-the-Art Review of Obstructive Versus Non-Obstructive Causes

**DOI:** 10.31083/j.rcm2307227

**Published:** 2022-06-24

**Authors:** Dinu V. Balanescu, Richard Bloomingdale, Teodora Donisan, Eric H. Yang, Purvi Parwani, Cezar Iliescu, Joerg Herrmann, Ivan Hanson

**Affiliations:** ^1^Department of Internal Medicine, Beaumont Hospital, Royal Oak, MI 48073, USA; ^2^Department of Cardiology, Beaumont Hospital, Royal Oak, MI 48073, USA; ^3^UCLA Cardio-Oncology Program, Division of Cardiology, Department of Medicine, University of California at Los Angeles, Los Angeles, CA 90095, USA; ^4^Department of Cardiology, Loma Linda University International Heart Institute, Loma Linda, CA 92354, USA; ^5^Department of Cardiology, The University of Texas MD Anderson Cancer Center, Houston, TX 77030, USA; ^6^Department of Cardiovascular Medicine, Mayo Clinic, Rochester, MN 55903, USA

**Keywords:** cardio-oncology, cancer, MINOCA, myocardial infarction in the absence of obstructive coronary artery disease

## Abstract

In patients with cancer, myocardial infarction (MI) has distinct features and mechanisms compared to the non-oncology population. Triggers of myocardial ischemia specific to the oncology population have been increasingly identified. Coronary plaque disruption, coronary vasospasm, coronary microvascular dysfunction, spontaneous coronary artery dissection, and coronary oxygen supply-demand mismatch are all causes of MI that have been shown to have specific triggers related to either the treatments or complications of cancer. MI can occur in the presence or absence of atherosclerotic coronary artery disease (CAD). MI with nonobstructive CAD (MINOCA) is a heterogeneous syndrome that has distinct pathophysiology and different epidemiology from MI with significant CAD (MI-CAD). Recognition and differentiation of MI-CAD and MINOCA is essential in the oncology population, due to unique etiology and impact on diagnosis, management, and overall outcomes. There are currently no reports in the literature concerning MINOCA as a unified syndrome in oncology patients. The purpose of this review is to analyze the literature for studies related to known triggers of myocardial ischemia in cancer patients, with a focus on MINOCA. We propose that certain cancer treatments can induce MINOCA-like states, and further research is warranted to investigate mechanisms that may be unique to certain cancer states and types of treatment.

## 1. Introduction

Myocardial infarction (MI) in cancer patients has distinct features and mechanisms compared to the general, non-oncology population. MI can broadly be categorized into MI due to coronary artery disease (MI-CAD) and MI in the absence of coronary obstructive disease (MINOCA). MI-CAD is the most common cause of MI in both cancer and non-cancer patients. Although traditional cardiovascular risk factors apply to both patients with and without cancer, the overall risk for MI in oncology patients is higher due to both cancer-related processes and anti-cancer therapies [1]. Even in the absence of cardiotoxic anti-cancer treatments, cancer patients can be found with high levels of cardiac biomarkers, suggesting subclinical myocardial damage of unclear etiology and associated with worse outcomes [2, 3]. MINOCA is a newly recognized heterogeneous syndrome that has distinct pathophysiology and epidemiology when compared to MI-CAD [4]. The prevalence of MINOCA among patients presenting with suspicion of acute MI was reported as high as 14% [5]. Recent data suggest that patients presenting with ST-elevation MI (STEMI) who have a history of cancer are more likely to have MINOCA rather than MI-CAD compared to patients with STEMI without an oncologic history (17% vs. 8%, respectively) [6]. Recognition of this condition and distinction from MI-CAD are essential, as MINOCA may be mis-diagnosed as non-cardiac, with significant cardiovascular management and outcome implications.

With the recent rapid rise of cardio-oncology, triggers of myocardial ischemia specific to the oncology population have been increasingly identified [7, 8, 9]. Coronary plaque disruption, coronary vasospasm, coronary microvascular dysfunction, oxygen supply-demand mismatch, and spontaneous coronary artery dissection (SCAD), are all causes of MI that have been shown to have specific triggers related to either the treatments or complications of cancer [10, 11, 12]. The multiple etiologies of MINOCA each portend different prognoses and require individualized management strategies [12]. Currently there is a paucity of data in the literature concerning MINOCA as a unified syndrome in oncology patients. The purpose of this review is to analyze the literature for studies related to known triggers of myocardial ischemia and infarction in cancer patients with a focus on MINOCA and propose that certain cancer states, and/or their treatments can induce MINOCA-like states.

## 2. Definitions

The Fourth Universal Definition of MI, issued by the Joint European Society of Cardiology (ESC), American College of Cardiology (ACC), American Heart Association (AHA), and World Heart Federation (WHF) Task Force, is widely accepted and used in clinical practice [13]. This most recent iteration of the universal definition of MI classifies troponin elevation as being due to acute ischemia (leading to myocardial infarction) and not acute ischemia driven (e.g., myocardial injury due to acute myocarditis).

**Fig. 1.**
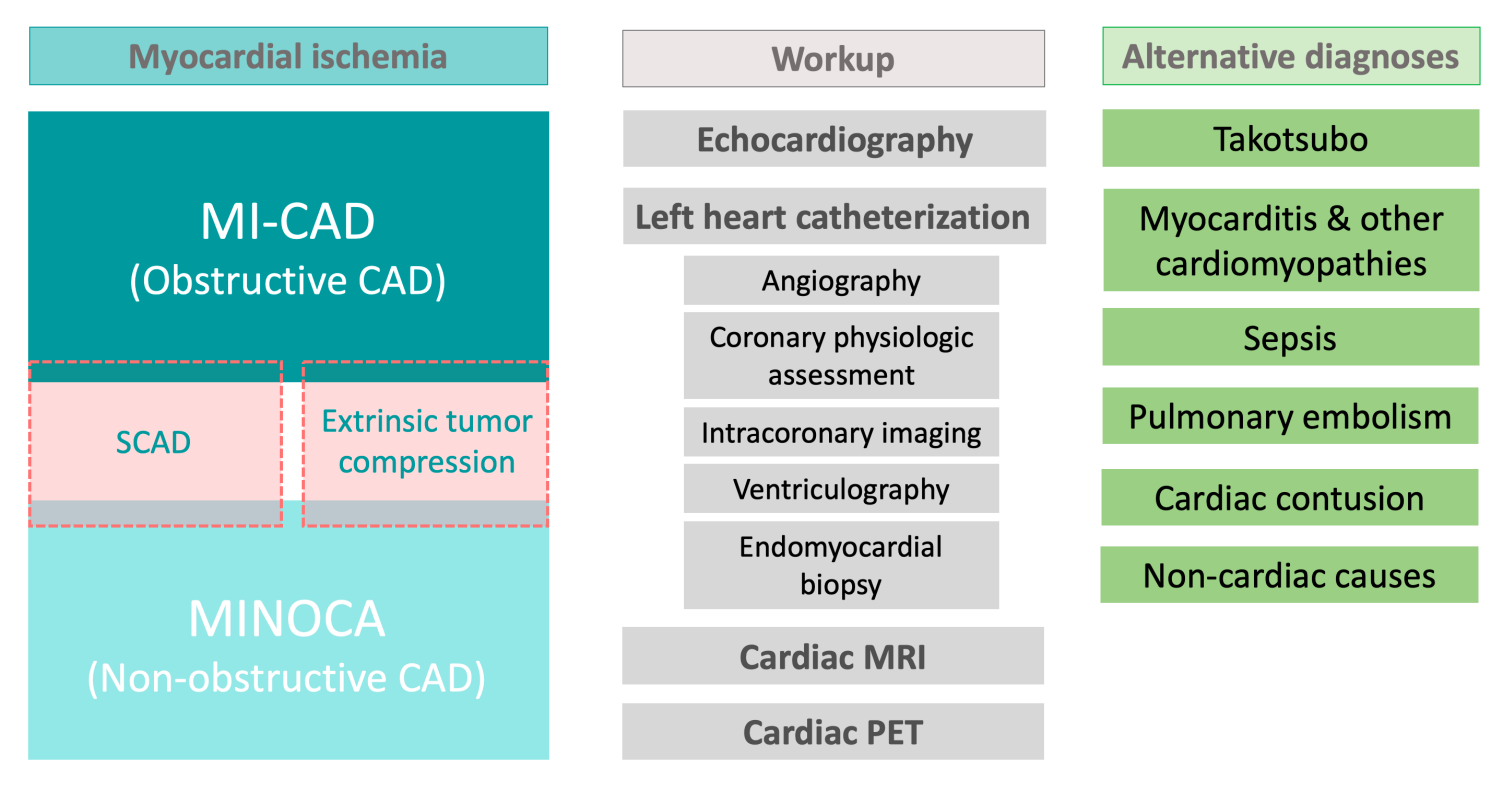
**Patients with cancer and ischemia may require extensive workup to differentiate between the various causes of obstructive and non-obstructive coronary artery disease and to exclude alternative diagnoses**. Abbreviations: CAD, coronary artery disease; MI-CAD, myocardial infarction with obstructive coronary artery disease; MINOCA, myocardial infarction with non-obstructive coronary artery disease; MRI, magnetic resonance imaging; PET, positron emission tomography scan; SCAD, spontaneous coronary artery dissection.

MINOCA is a recently described entity that can broadly be defined by these criteria: (1) acute MI according to the Fourth Universal Definition of MI; (2) exclusion of missed obstructive coronary disease (e.g., coronary emboli/thrombi, coronary dissection); (3) no coronary lesions ≥50% in a major epicardial vessel; (4) no coronary lesions with FFR <0.8; (5) no other identifiable cause for the presentation [12, 14]. MINOCA can be considered as a working diagnosis, when known causes of elevated troponin are excluded and our current diagnostic capacity has been reached. Initial studies of MINOCA considered the complete absence of coronary disease as a diagnostic necessity [15]. It is currently generally accepted that the diagnosis can be established even in the presence of moderate coronary disease (i.e., obstructing <50% of the lumen) [4]. Echocardiography, ventriculography, and cardiac magnetic resonance (CMR) imaging should be part of the work-up when assessing for MINOCA to comprehensively search for etiologies that may mimic the syndrome (Fig. 1). CMR is an essential diagnostic tool in these patients since it can evaluate the non-ischemic and ischemic etiology once the working diagnosis of MINOCA is established. Data on the functional assessment of coronary lesions in MINOCA are sparse. It was noted that a significant number of lesions in the 30%–50% range by angiography are functionally hemodynamically significant on fractional flow reserve (FFR) or cardiac positron emission tomography (PET) and responsible for MI-CAD [16, 17]. There are no data on instantaneous free-wave ratio in MINOCA. A special category of cancer patients undergoing therapy with immune checkpoint inhibitors (ICI) and possible myocarditis can have indeterminate CMR findings [18] and in some cases require endomyocardial biopsy (EMB), which can be challenging in the acute coronary setting where the addition of antiplatelet and anticoagulation increases the bleeding risk and the subsequent risk of perforation and tamponade. Intracoronary imaging and functional assessment should also be used to identify specific causes of MINOCA once an ischemic etiology is established via CMR [12].

Despite MINOCA having an overall 1-year mortality rate of 4.7%, data on the impact of distinct management strategies based on the specific diagnosis is lacking in the literature [19]. This may be due to MINOCA being a relatively heterogeneous entity, with various etiologies included in the disease spectrum, with an evolving definition. The diagnosis of MINOCA can be difficult, given that many chemotherapies and targeted therapies can cause non-specific cardiac biomarker elevations and/or EKG or echocardiographic abnormalities, which can also be compounded by other concomitates states associated with complications of cancer and its therapies (e.g., sepsis, hypovolemia). The overlap of these states with MINOCA is unclear. Scientific statements from the AHA and ESC proposed algorithms for diagnosing MINOCA, which specifically exclude from the MINOCA spectrum non-ischemic causes of troponin elevation, such as cardiac contusion, coronary emboli/thrombi, Takotsubo syndrome (TTS) and other cardiomyopathies, and myocarditis [12, 14] requires a comprehensive work-up which is infrequently performed.

## 3. Etiology and Mechanisms of Myocardial Infarction in Cancer Patients

Based on the above definitions, we considered the following specific causes of MI-CAD and MINOCA: coronary plaque disruption, epicardial coronary vasospasm, coronary microvascular dysfunction, coronary oxygen supply-demand mismatch (type 2 MI), and SCAD [19]. The epidemiology of these specific causes of MI is different in cardio-oncology patients compared to the general cardiovascular population, given the unique risk profile of cancer patients and various cardiotoxic anti-cancer therapies. As such, we performed a literature search for studies addressing each of these situations in cancer patients. A special note is made regarding TTS and myocarditis, two entities that were initially considered triggers of MINOCA given their very similar presentation to non-ST elevation MI (NSTEMI), but which have been excluded from the MINOCA spectrum in the most recent societal statements. TTS has a proposed ischemic mechanism that includes coronary vasospasm and microvascular dysfunction (in addition to catecholamine-induced toxicity) [20]. Furthermore, TTS is more prevalent in cancer patients presenting with apparent NSTEMI compared to the general population, with approximately 10% of patients with cancer who exhibited clinical characteristics of NSTEMI being ultimately found with TTS [21, 22]. Cancer patients are also exposed to various agents that may induce both acute coronary syndromes and myocarditis, which have similar initial presentations [23]. We also considered extrinsic coronary compression as a mechanism of myocardial ischemia in cancer patients. Given these special considerations in cardio-oncology, coronary compression, TTS, and myocarditis will also be addressed in this review as syndromes that specifically need to be considered in cancer patients presenting with apparent NSTEMI or MINOCA.

### 3.1 Coronary Plaque Disruption

Cancer and coronary artery disease (CAD) frequently coexist due to shared risk factors, an increasing pool of cancer survivors that age, and increased recognition of the cardiovascular toxicity of anti-cancer therapies during active cancer treatment and in cancer survivors [24, 25, 26]. Furthermore, the pro-inflammatory and pro-thrombotic nature of malignancies increases the risk for endothelial damage and progression of atherosclerotic disease in an oncology population, in addition to the endothelial dysfunction/injury from chemo-, immune- or radiation therapy [24, 27, 28]. Up to 15% of patients presenting with acute coronary syndromes have either active or a history of cancer [29]. Cancer can be considered a risk factor for CAD, with increasing data suggesting the direct cause-and-effect relationship between cancer and CAD [28]. Supply-demand mismatch in stable CAD is common in cancer patients due to a high risk of anemia, sepsis, tachycardia, and hypovolemia, in this population, although this represents a different mechanism and will be discussed separately.

Similar to MI-CAD, the fundamental atherosclerotic mechanism of MINOCA is coronary plaque disruption [30, 31]. Plaques prone to disruption (“vulnerable”) are generally angiographically mild [32]. More commonly in MINOCA than MI-CAD, plaque disruption occurs in positively-remodeled lesions, i.e., lesions expanding outward from the coronary wall instead of obstructing the lumen, thus not evident on regular coronary angiography [32]. These lesions require definitive assessment with intracoronary imaging, either intravascular ultrasound (IVUS) or, preferred if available, optical coherence tomography (OCT) [31]. Cancer patients appear to have accelerated vascular aging as reflected by increased calcium scores when compared to non-cancer patients, which, in turn, also places them at a higher risk for acute MI [24], be it MINOCA or MI-CAD.

Plaque disruption includes the following 3 mechanisms: plaque rupture, plaque erosion, and calcified nodules [12] all with a common pathophysiologic endpoint of acute MI via thrombosis. The risk of venous thrombosis in cancer patients has been extensively studied and is well-established, however, recent evidence suggests that the risk of arterial thrombosis is currently underestimated [27, 33, 34]. Cancer patients have been shown to have elevated levels of platelet activation markers, such as platelet factor 4, P-selectin, and soluble CD40 ligand [35]. Cancerous cells have also been shown *in vitro* to directly induce platelet activation and aggregation [36]. The mechanism, generally termed tumor cell-induced platelet aggregation, involves several molecular pathways, including ADP, thrombin, tissue factor, metalloproteinases, thromboxane $A_{2}$, and VEGF [36, 37]. There is increasing evidence supporting the involvement of neutrophil extracellular traps (NETs) in cancer-induced thrombosis, which may clinically present with elevated serum troponins in ischemic stroke patients [38, 39]. In a small 2016 case series on 31 patients assessing causes of troponin elevation in patients with ischemic stroke, the only significant difference between patients with and without elevated serum troponin was the presence of active cancer [39]. Three of the patients with troponin elevation in this study were diagnosed with active cancer post-mortem. On autopsy, these patients were all found with only mild coronary atherosclerosis, no thrombotic occlusions, but with widespread coronary microvascular thrombosis, disseminated focal areas of myocardial damage, and presence of NETs. Although the main focus of the study was ischemic stroke, the authors reported NET-associated myocardial arterial microthrombosis in the coronary vasculature due to cancer as a cause of troponin elevation in the context of no epicardial coronary disease [39]. Interestingly, the risk of arterial thrombosis persists even in the setting of chronic thrombocytopenia (a frequent comorbidity in the course of malignancies) [40].

In addition to direct cancer-induced mechanisms of thrombosis, there is also a strong association between anti-cancer therapies and a risk of arterial thrombosis [41] (Table 1). This includes numerous classes of chemotherapeutic agents, immunotherapies, and radiation therapy. For example, Animal and human studies showed that radiation-therapy leads to accelerated atherosclerosis and vulnerable plaque development [42].

**Table 1.** **Mechanisms of cancer treatment-induced myocardial ischemia (MI-CAD + MINOCA)**.

	Plaque disruption and prothrombotic effects	Vasospasm	Microvascular dysfunction
Alkylating agents (cisplatin, cyclophosphamide)	+	+	+
Antimetabolites (5-fluorouracil, capecitabine)	-	+	+
Anthracyclines (doxorubicin)	-	+	+
Microtubule-binding agents (paclitaxel)	-	+	+
Antitumor antibiotics (bleomycin)	+	+	
Plant alkaloids (vincristine, etoposide)	+	+	+
Proteasome inhibitors (bortezomib, carfilzomib)		+	
Anti-VEGF (bevacizumab)	+	+	+
TKI inhibitors (e.g., ponatinib, sorafenib, sunitinib, axitinib, pazopanib)	+	+	+
Immune checkpoint inhibitors (e.g., pembrolizumab, nivolumab, atezolizumab, ipilimumab)	+	-	-
CAR T-cell therapy	+	-	-
Radiotherapy	+	+	+

*Abbreviations*: CAR, chimeric antigen receptor; TKI, tyrosine kinase inhibitor; VEGF, vascular endothelial growth factor. Adapted from Herrmann J, Yang EH, Iliescu CA, Cilingiroglu M, Charitakis K, Hakeem A, *et al*. Vascular Toxicities of Cancer Therapies: The Old and the New–An Evolving Avenue. Circulation. 2016;133(13):1272-89.

Although there are no studies currently in the literature specifically assessing MINOCA in cancer patients, evidence regarding this syndrome and arterial thrombosis in cardio-oncology may be inferred from several works. A case report of a patient who developed sudden cardiac death while on cisplatin, bleomycin, and etoposide for testicular cancer suggested demonstrated an acute fibrin thrombus on autopsy overlying mild atherosclerotic disease [43]. Cisplatin in particular may be related to this effect, as it has been described on forearm venous occlusion plethysmography to induce acute and transient endothelial toxicity [44]. A recent study from Israel analyzed consecutive patients who underwent coronary angiography for clinically defined acute MI and who were found without obstructive CAD [45]. The study included 174 such patients who were matched with a control group of 348 adults with MI-CAD. The authors identified MINOCA presenting as NSTEMI to be a significant independent risk factor for occult malignancies (odds ratio 4.6) and attributed this effect mainly to arterial thromboembolism, although they couldn’t definitively rule out other of the known triggers of MINOCA [45]. Another recent case-control analysis of coronary angiography findings in 240 cancer patients and 240 non-cancer patients identified a lower burden of angiographically-detectable coronary disease in the cancer group [46]. A significant limitation of that analysis was the inclusion of all patients undergoing coronary angiography without adjusting for indications for cardiac catheterization. Although the authors concluded that this “cancer paradox” may be due to cancer patients being referred for coronary angiography for indications other than CAD suspicion, and there’s no analysis of the prevalence of patients who had acute MI criteria. These findings also raise the hypothesis of a higher prevalence of MINOCA in the cancer group. A landmark study by Navi *et al*. [34] assessing 280,000 cancer patients in the Surveillance, Epidemiology, and End Results (SEER) database matched each patient to patients from the Medicare database. The authors identified MI via ICD codes, including numerous possible forms such as plaque rupture, embolism, vasospasm, and other forms of thrombosis. A diagnosis of cancer carried a significant hazard ratio (HR) of 2.2 for arterial thrombotic events and of 2.9 for MI. Of note, the authors excluded patients with a diagnosis of CAD one year prior to the cancer diagnosis. However, the SEER study did not include angiographic data, nor did it analyze specific triggers of MI, so it is unknown how many of the included patients with myocardial ischemia had MINOCA versus other forms of MI.

Immune checkpoint inhibitors (ICI) are increasingly and successfully used in the treatment of numerous cancers. These agents are associated with a range of immune-related adverse events (iRAEs), of which cardiotoxicity is among the most severe. Although the main focus in the literature with regards to cardiotoxicity has been on ICI-induced myocarditis, recently, multiple reports have been published suggesting a direct causal effect of ICI on coronary plaque disruption [47, 48, 49]. In a recent study of 1215 patients with cancer who received ICI, approximately 1% of patients developed either myocardial infarction or an ischemic stroke within 6 months of ICI treatment [50]. The same incidence of arterial thrombotic events after ICI therapy was described in a systematic review of 10,106 subjects [51]. The underlying mechanism appears to be a change in atherosclerotic inflammatory cell composition triggered by ICI [52]. Given these observations and the ubiquitous use of ICI, these agents should be recognized as potentially linked to MI and/or MINOCA, although further study is warranted.

Clonal hematopoiesis of indeterminate potential (CHIP) has recently emerged as an independent risk factor for CAD [53]. Mutations seen in CHIP are also seen in certain hematologic cancers, such as myelodysplastic syndromes and acute myeloid leukemia [54]. Patients with CHIP have a 10-time higher risk of developing a hematologic malignancy compared to those without CHIP [53]. The precise mechanism through which CHIP induces atherosclerotic disease is unclear. Although there are no reports currently of CHIP-associated acute MI, clinicians should be aware of CHIP as a causative agent for atherosclerotic disease.

Data regarding MINOCA secondary to non-hemodynamically significant coronary atherosclerosis in cancer patients is extremely limited. What is clear is that cancer patients are at increased risk for plaque disruption and arterial thrombosis, which increases risk of both MI-CAD and MINOCA. Further studies to advance the understanding of arterial thrombosis leading to MINOCA in cancer patients is essential to optimize management and develop preventive strategies, particularly in patients receiving thrombogenic anti-cancer therapies.

### 3.2 Epicardial Coronary Vasospasm

Coronary artery spasm (CAS) is an important cause of MINOCA, described in up to 46% of MINOCA patients [55]. Intense CAS may be significant enough to impede blood flow and cause myocardial ischemia. The diagnosis may be missed on coronary angiography, as the spasm may resolve before the procedure. Vasospasm can occur both in the absence or the presence of CAD, as atherosclerosis may precipitate vasospasm [56]. Definitive diagnosis requires provocative testing, the current standard being high-dose intracoronary acetylcholine boluses followed by coronary angiography. Although CAS may occur without apparent triggers, several anti-cancer therapies have been well documented to induce infarction by vasospasm.

The classic chemotherapies extensively described as inducing CAS are the fluoropyrimidines 5-fluorouracil (5-FU) and its oral prodrug, capecitabine. The pathophysiology of 5-FU cardiotoxicity is multifactorial. Histology studies found changes such as pan-cardiac inflammatory changes, coronary arterial spasm, hemorrhagic infarction of ventricular walls, myocardial interstitial fibrosis, disseminated myocardial necrosis, and coronary microthrombosis [57, 58]. These changes were found to be dependent on treatment dose and schedule. Between 1–19% of patients receiving 5-FU develop chest pain attributed to CAS [59, 60], irrespective of pre-existing cardiovascular disease [61, 62]. This effect is amplified in the setting of simultaneous administration of other chemotherapeutic agents, especially leucovorin or cisplatin [62, 63, 64]. The significant range may be attributed to differences between administration methods, underlying CAD, or use of other anti-cancer treatments. A prospective study on 102 unselected patients treated with 5-FU were followed with ECG, echocardiography, and radionuclide ventriculography at baseline and 3 months from starting 5-FU. Nineteen of the 102 patients developed severe chest pain, with EKG changes suggestive of myocardial infarction [65]. Six of them underwent coronary angiography. None of them were found with significant CAD. The authors of these study report that cardiac enzymes were measured initially negative in these patients. However, multiple reports of troponin elevation with normal coronary angiography in patients receiving 5-FU undergoing extensive cardiac assessment have been published [66, 67]. A 2009 systematic review of fluoropyrimidine-associated cardiotoxicity describes a 12% prevalence of increased cardiac enzymes [62]. These findings are consistent with MINOCA. Further angiographic data in patients with suspected 5-FU or capecitabine cardiotoxicity are limited to individual case reports. These reports consistently show the lack of significant coronary artery disease [66, 68, 69, 70, 71, 72]. The mechanism best supported by both preclinical and clinical data for these findings is CAS related to endothelial dysfunction [9, 73, 74]. Although inconsistently reported, CAS and brachial artery vasoconstriction have been directly demonstrated during angiography [75, 76]. The risk of recurrence of such ischemic events with 5-FU rechallenge is as high as 90% [77]. This effect is “cross-reactive” with cisplatin, although there have been reports of successful capecitabine use following 5-FU cardiotoxicity [78]. Therefore, special considerations are needed when considering 5-FU rechallenge in patients with 5-FU-induced MINOCA via CAS. If no other anti-cancer regimen is reasonable, several strategies may be attempted, such as bolus instead of 5-FU infusion [79, 80], or giving low-dose aspirin and a calcium-channel blocker and long-acting nitrate at least 72 hours prior to 5-FU administration (although this approach is mainly based on consensus rather than evidence-backed) [81].

A number of different chemotherapies are also associated with CAS. Cisplatin has been associated with numerous vascular toxicities. There are few reports of MI with troponin measurements and documented coronary angiography following cisplatin administration. These reports attributed the ischemic event to CAS [82, 83, 84, 85, 86]. Troponin elevation was inconsistently present, but coronary angiography recurrently showed no significant CAD, consistent with MINOCA. Notably, one report documented that acetylcholine provocation induced severe coronary vasospasm associated with chest pain and significant ST elevation [82]. Cisplatin-induced MINOCA via CAS may occur early during the treatment regimen or delayed for years after completing cisplatin treatment [82]. Since cisplatin has been associated with type 1 MI due to coronary thrombosis, angiographic assessment (optimally invasive) is advised in patients treated with this agent presenting with apparent ACS [11].

Vasospasm has been proposed as the underlying mechanism of taxane-induced ACS. Paclitaxel is an antimicrotubule agent which has been linked with ACS, acute heart failure, bradycardia, and cardiovascular mortality [87]. Paclitaxel-induced MI is a rare adverse event, estimated to occur in ~0.26% of cases [88]. There are several case reports of paclitaxel-induced MI, with inconsistent troponin elevation, transient ST-elevation, demonstrated coronary vasospasm, and both obstructive and non-obstructive CAD [88, 89, 90]. The proposed mechanism of taxane-induced vasospasm is reduced calcium release in the sarcoplasmic reticulum [91].

Angiogenesis inhibition is currently expanding as a cancer treatment strategy. Vascular endothelial growth factor (VEGF) inhibitors are increasingly being used as part of this strategy. Physiologically, VEGF is essential to normal endothelial function and maintaining hemostasis and thrombosis [87]. Low levels of VEGF have been associated with increased cardiovascular mortality in patients with known or suspected CAD [92, 93]. VEGF inhibitors include the monoclonal antibodies bevacizumab and regorafenib and the small molecule tyrosine kinase inhibitors (TKIs) such as ponatinib, sorafenib, sunitinib, axitinib, and pazopanib. Both bevacizumab and small molecule TKIs have been strongly associated with arterial thrombotic events [94], however, only small molecule TKIs have been found to also induce vasospasm. Data related to TKI-induced CAS is limited to case reports [95, 96]. Troponin elevations in cases of non-obstructive CAD were not consistently reported, making MINOCA an unclear entity related to TKIs [95, 96, 97]. In patients with known CAD, performing a stress test and treatment with aspirin and a statin prior to and during TKI therapy is reasonable, as well as treating with calcium channel blockers should vasospasm be identified.

More recently, proteasome inhibitors have been linked to CAS [98]. Bortezomib and carfilzomib are proteasome inhibitors used in the treatment of multiple myeloma. Although these agents are strongly associated with acute heart failure thus leading to MINOCA indirectly via type 2 MI, they have also been linked to CAS. Murine studies on carfilzomib suggest that this agent impairs vasodilation through an endothelium-dependent mechanism and increases the spasmogenic effect of other agents [98]. Bortezomib is another frequently used proteasome inhibitor which has been shown to induce CAS in humans [99, 100]. Vasospasm was mainly described in the left coronary system, most frequently in the left anterior descending artery. Calcium channel blockade inconsistently improved symptoms and recovery of cardiac function. *In vitro*, nifedipine was less effective than nitroglycerin at inhibiting proteasome-inhibitor-induced vasospasm, which suggest using nitrates in this setting as opposed to calcium channel blockers [98].

Symptomatic CAS has also been reported in patients undergoing radiation therapy (RT) [101, 102]. Several mechanisms have been hypothesized for RT-induced CAS, such as radiation-induced pericarditis and radiation-induced vasculitis or arteritis [103]. More recently, a direct effect of RT on vascular reactivity has been described. RT impairs endothelium-dependent vasorelaxation by decreasing nitric oxide availability, an effect which may persist for years [104, 105, 106]. RT-induced CAS appears to be refractory to vasodilators and may improve with glucocorticoids [102].

CAS is one of the most common mechanisms of MINOCA in cancer patients. Although most frequently precipitated by chemotherapy, chronic inflammation and oxidative stress intrinsic to the cancer status predispose patients to this adverse event. Troponin elevation may or may not occur in cases of chemotherapy-induced CAS and vasospasm may not be directly identified on coronary angiography. However, MINOCA in this setting should be recognized and prophylactic measures should be implemented when agents known to cause CAS are being considered as part of cancer therapy. Calcium channel blockers are first-line therapies, although not always resolving symptoms or preventing recurrences. Further studies into the mechanisms and effective prophylactic and therapeutic measures of CAS-induced MINOCA in cancer patients are required.

### 3.3 Coronary Microvascular Dysfunction

The coronary microcirculation is not readily visualized on routine clinical imaging modalities, despite accounting for >70% of coronary resistance in the setting of no obstructive CAD [107]. Coronary microvascular dysfunction (CMD) has been described in 30–50% of cases presenting with chest discomfort and non-obstructive CAD on coronary angiography [108]. The criteria for microvascular angina were standardized by the Coronary Vasomotion Disorders International Study Group and they include: (1) symptoms suggestive of myocardial ischemia; (2) objective evidence of myocardial ischemia; (3) absence of obstructive CAD by angiography or FFR; (4) confirmation of reduced coronary blood flow reserve and/or inducible microvascular spasm [108]. Although microvascular angina is not equivalent to MI, it may progress to MINOCA if undiagnosed and untreated. Only a minority of patients with CMD will have regional wall motion abnormalities on echocardiography [108], highlighting the need for multimodality imaging, with cardiac PET playing a role in identifying these patients. CMD is a predictive factor for other CVD, in particular heart failure with preserved ejection fraction. CMD has also recently been associated with a two-fold increased risk of developing solid-tumor cancer, suggesting the need for increased awareness of CMD in cardio-oncology [109].

The mechanisms through which anti-cancer therapies cause CMD are similar to those responsible for epicardial coronary disease with endothelial dysfunction playing a central role, resulting from decreased nitric oxide production, oxidative stress with release of reactive oxygen species, and increased endothelin-1 and angiotensin II release and production. Other mechanisms that also lead to CMD in cancer patients are atherosclerosis, thrombosis, microvascular CAS, hormonal effects, and autonomic dysfunction. Anti-cancer treatments have been shown to induce CMD via the above mechanisms.

VEGF inhibitors have been associated with arterial thrombotic events and CAS, as mentioned above. The abnormal vasoreactivity triggered by these agents may be even more significant on the coronary microcirculation than the epicardial coronaries [110]. Bevacizumab is a VEGF inhibitor used in multiple cancers. All patients with known heart failure should undergo coronary angiography prior to initiating bevacizumab to exclude CAD [111]. The mechanism of CMD induced by VEGF inhibitors is decreased nitric oxide production impairing endothelium-mediated vasodilation [112] and increased endothelin-1 and angiotensin II production [113]. Although bevacizumab cardiotoxicity is well-recognized and arterial thrombotic events are a major concern [114], data on bevacizumab-induced MI are scarce. Murine models showed a twofold increase in serum troponin levels in mice following a 3-week treatment with bevacizumab, as well as evidence of myocardial necrosis as early as 2 weeks of treatment [115, 116]. Human data on bevacizumab-induced MINOCA is limited to case reports which also include coronary angiography data [117, 118]. Although no mechanism has been clearly identified, given the toxicity profile of bevacizumab, coronary microthrombosis is a reasonable hypothesis as the underlying mechanism of these events, although further studies are needed. Nicorandil, a vasodilator agent, was successfully used to treat microvascular angina associated with bevacizumab [118]. Third-generation TKIs have been notoriously associated with rapidly-progressive vasculopathy. Unique to sunitinib is the observation that in mice, it induced rarefication of microvascular pericytes without changing capillary density, with subsequent development of microvascular dysfunction and impaired coronary flow reserve [119]. Ponatinib has been shown to cause microvascular coronary angiopathy by inducing von Willebrand factor-mediated platelet-endothelial adhesion [120]. Myocardial contrast echocardiography was used as a rapid bedside diagnosis of coronary microvascular disease in cases of suspected ponatinib-induced acute MI with elevated troponin [121].

Radiation-induced CAD (RI-CAD) is an important cause of morbidity in patients who undergo RT with mediastinal involvement. The risk of CAD in such patients is increased as much as 2.5 times compared to patients without radiation therapy exposure [122]. RT with incidental cardiac exposure can disrupt the capillary endothelial structure and cause direct myocyte injury, leading to episodes of ischemia, collagen deposition, and fibrosis [123, 124]. Biochemically, the result is an increase in transforming growth factor-beta, which leads to a pro-thrombotic and pro-inflammatory state which predisposes to accelerated atherosclerosis. This effect may present even in the absence of prior CAD or traditional cardiovascular risk factors, although the presence of these elements shortens the time to the development of atherosclerosis [125]. The dose of radiation is linearly associated with the risk of RI-CAD [126]. *In vitro*, this effect was not augmented by trastuzumab, which may translate to the use of trastuzumab without concern for microvascular dysfunction [127]. The evaluation of patients with RI-CAD is similar to ACS, however, in patients found with non-obstructive CAD, further work-up with functional testing or cardiac MRI is advised. If CMD is diagnosed, aggressive cardiovascular risk factor management should be immediately started with close follow-up.

In addition to the VEGF inhibitors and RT, recently, doxorubicin has been shown *ex vivo* to induce significant impairment of coronary arteriolar function in vessel samples collected from adults undergoing cardiopulmonary bypass surgery [128]. Interestingly, this effect was insignificant in pediatric coronary microcirculation.

Although there are few angiographic and serologic data regarding CMD as a cause of MINOCA in cancer patients, by definition CMD is part of the MINOCA spectrum. Doxorubicin, VEGF inhibitors, and RT are all associated with significant morbidity related to CMD that may progress to overt MINOCA. Chest pain or anginal equivalents should not be dismissed as non-cardiac in patients receiving these therapies with unremarkable coronary angiography and microvascular angina should be considered as a leading diagnosis.

### 3.4 Coronary Oxygen Supply-Demand Mismatch

According to the Fourth Universal Definition of Myocardial Infarction, type 2 MI (T2MI) is the result of myocardial oxygen supply-demand mismatch [13]. In patients diagnosed with MI, T2MI is up to 48% prevalent [129]. In patients with T2MI, MINOCA can be diagnosed when a plausible trigger for MI exists in the absence of angiographic or imaging evidence that would suggest another diagnosis [12]. One of the most common causes of T2MI is tachyarrhythmia-associated acute MI, with other potential causes being anemia, hypotension, or thyrotoxicosis [130]. Given the nature of malignant disease, these conditions are prevalent in the cancer population, placing them at high risk for T2MI. However, there are few data on cancer patients with T2MI, as invasive assessment is generally deferred in comorbid patients with sufficient clinical evidence of a T2MI with low suspicion of obstructive coronary disease. Cancer patients with T2MI have been shown to have worse overall survival than those with type 1 MI, potentially related to a higher burden of non-cardiac comorbidities, although etiologic mortality data has not been reported [131, 132]. A retrospective cohort study from the Mayo Clinic of patients with active hematologic malignancies diagnosed with ACS found that 67% of studied patients who underwent coronary angiography had T2MI, consistent with MINOCA. Only 17.5% of patients with NSTEMI in this study underwent coronary angiography, with T2MI suspected in the majority of cases with invasive work-up deferred [133]. Differentiating clinically between T2MI, other forms of cardiotoxicity, and the pure definition of MINOCA in cancer patients, can be difficult and further research is needed regarding the optimal management of these cases.

### 3.5 Spontaneous Coronary Artery Dissection

Spontaneous coronary artery dissection (SCAD) is a nontraumatic, nonatherosclerotic cause of ACS and sudden cardiac death [134]. SCAD was thought to be very rare, including in cancer patients, but recent efforts found a higher prevalence than previously believed and provide a better understanding of this clinical entity [135]. SCAD has typically been described in middle aged women (87–95% of SCAD), but it can occur anytime from late teens to the ninth decade of life [135]. The mechanism for acute MI in SCAD is the development of a hematoma within the intima or between the intima and media, compressing the coronary true lumen. The hematoma is thought to arise in two ways: an endothelial-intimal disruption creates a “flap” through which blood can enter the sub-intimal space—the “inside-out” hypothesis; and possible de novo disruption of vasa vasorum in the media, causing a hematoma without any communication with the true lumen—the “outside-in” hypothesis [136, 137]. SCAD can lead to MINOCA in cases where the false lumen is nonobstructive or in acute intracoronary thrombosis in the absence of prior significant atherosclerotic disease. SCAD may require intracoronary imaging techniques for definitive diagnosis [138]. The mechanism of SCAD is unclear, but thought to be due to an intrinsic vascular vulnerability superimposed with an acute catecholamine surge (i.e., emotional stress, physical activity, medications) [139]. SCAD seems to occur independently of atherosclerosis, not being associated with conventional cardiac risk factors. SCAD has been reported to have various triggers [140], some of which are not usually associated with acute MI, such as emotional or physical stress [139, 141].

There are few case reports of SCAD occurring in cancer patients undergoing chemotherapy with 5-FU and/or cisplatin [142, 143, 144], bone marrow transplant for chronic lymphocytic leukemia [145], and in patients without active cancer treatment [146]. None of these patients had significant atherosclerotic disease on coronary angiography. Because of the overwhelming majority of SCAD cases presenting in women, sex hormones have been studied to assess any pathogenic mechanism. It is unclear what this mechanism is or if sex hormones are involved, as SCAD can occur in pregnant, postpartum, nulliparous, multiparous, and post-menopausal women [147, 148], and contraceptive and postmenopausal hormone use are similar to general population [149, 150]. There have been no studies regarding the relationship between hormone-altering cancer therapies (e.g., in breast, endometrial, testicular, or prostate cancers) and SCAD. There are no reports of intracoronary imaging used in cancer patients with SCAD, so it is reasonable to hypothesize that there are a number of MINOCA cases caused by SCAD that remain undiagnosed. Thorough intracoronary imaging should be considered in suspected cases of MINOCA.

### 3.6 Special Considerations: Extrinsic Coronary Compression, Takotsubo Syndrome, Myocarditis

In addition to the above specific causes of myocardial ischemia, in cancer patients, several special considerations should be noted.

Patients with intrathoracic masses are at risk for acute MI from extrinsic coronary compression. Although this etiology hasn’t been proposed as a cause of MINOCA in societal guidelines, it may conform to the definition of MINOCA. Both primary and metastatic tumors and both cardiac and extracardiac masses may compress on any coronary artery. Small epicardial branches have been more frequently involved, although there have also been reports of left main and proximal left anterior descending artery involvement [151, 152]. Patients may present with both STEMI and non-STEMI and may be found with both angiographically significant and non-significant stenoses [153, 154]. Coronary angiography may show completely normal coronaries in young patients, leading to suspicion of CAS as the etiology of MINOCA. However, further testing with intracoronary imaging, cardiac CT, or CMR may be warranted, particularly in young patients with clear MINOCA and otherwise no cardiovascular history or risk factors, which may reveal intramyocardial metastases of mediastinal tumors [153]. Cardiac primary tumors or extracardiac malignancies with secondary cardiac determination with or without coronary compression have abnormal cardiac biomarkers, ECG, and should be differentiated from other causes of ACS and MINOCA.

Special situations worth noting in cardio-oncology patients are TTS and myocarditis. Although these syndromes are no longer considered MINOCA per the most recent societal documents, TTS and myocarditis have a high prevalence and unique triggers in cardio-oncology patients, making them worth noting as causes of ischemia-like presentations with no obstructive coronary disease. In fact, as much as 20% of cancer patients presenting with suspicion of non-STEMI are ultimately diagnosed with TTS and up to 30% of patients initially diagnosed with MINOCA based on coronary angiography are ultimately found with myocarditis following advanced non-invasive imaging [22, 155]. The exact pathogenic mechanism of TTS is still unknown and may in fact be related to other causes of MINOCA, such as CAS and CMD [156]. Emotional stress related to the cancer diagnosis and treatment, the pro-inflammatory state of malignancy, and chemoradiation may all precipitate TTS [157]. Numerous classes of chemotherapeutic agents have been recognized as triggering TTS and myocarditis, including novel immunotherapies such as lenalidomide and immune checkpoint inhibitors [23, 158, 159, 160]. Patients treated with these agents may be misdiagnosed as having MINOCA while they have an unrecognized myocarditis. There are no prospective clinical trial data to guide management of cancer-related TTS or myocarditis. TTS is generally treated with guideline-directed medical therapy for heart failure with reduced ejection fraction regardless of apparent trigger, although outcome data does not show a clear benefit of any regimen [156, 161]. Distinguishing between MINOCA and cardiomyopathies or inflammatory syndromes such as myocarditis can be difficult and more investigation is required to determine optimal management.

## 4. Conclusions

Ischemic assessment in cancer should involve troponin assessment in combination with other cardiac biomarkers and novel imaging modalities given the complex and heterogeneous pathophysiology of cancer. MI-CAD and MINOCA in cardio-oncology patients have unique triggers, each portending different management strategies and prognoses. Multiple chemotherapeutic regimens may trigger MI, with the main mechanisms being coronary thrombosis, CAS, and CMD. Although further studies are needed in investigating the mechanisms of MI for individual cancer treatments, integrating the term MINOCA in classifying forms of cardiotoxicity may be useful throughout the diagnostic process. Historically the original “type I/type II” cardiotoxicity paradigm was used to define major types of cardiotoxicity [162] but was oversimplified and primarily focused on the mechanisms of cardiotoxicity of anthracyclines and anti-HER2 treatments. However, as previously outlined in this review, there are both historical and novel cancer treatments that may induce MINOCA-like states which raises the potential applicability of this term as a form of cardiotoxicity. Further study of mechanisms and noninvasive and invasive diagnostic strategies are needed to further understand the unique mechanistic aspects of MINOCA syndromes in specific cancer treatments and/or biology. The diagnosis of MINOCA implies the presence of true myocardial ischemia and causes of non-ischemic myocardial injury should be excluded. The dynamics of cardiac biomarkers, intracoronary imaging, and multimodality imaging should be considered as part of comprehensive cardiovascular work-up in cancer patients presenting with ACS and non-obstructive CAD on coronary angiography.

## References

[1] Potts JE, Iliescu CA, Lopez Mattei JC, Martinez SC, Holmvang L, Ludman P (2019). Percutaneous coronary intervention in cancer patients: a report of the prevalence and outcomes in the United States. *European Heart Journal*.

[2] Pavo N, Raderer M, Hülsmann M, Neuhold S, Adlbrecht C, Strunk G (2015). Cardiovascular biomarkers in patients with cancer and their association with all-cause mortality. *Heart*.

[3] Finke D, Heckmann MB, Wilhelm S, Entenmann L, Hund H, Bougatf N (2022). Coronary artery disease, left ventricular function and cardiac biomarkers determine all-cause mortality in cancer patients-a large monocenter cohort study. *Clinical Research in Cardiology*.

[4] Safdar B, Spatz ES, Dreyer RP, Beltrame JF, Lichtman JH, Spertus JA (2018). Presentation, Clinical Profile, and Prognosis of Young Patients with Myocardial Infarction with Nonobstructive Coronary Arteries (MINOCA): Results from the VIRGO Study. *Journal of the American Heart Association*.

[5] Ballesteros-Ortega D, Martínez-González O, Gómez-Casero RB, Quintana-Díaz M, de Miguel-Balsa E, Martín-Parra C (2019). Characteristics of patients with myocardial infarction with nonobstructive coronary arteries (MINOCA) from the ARIAM-SEMICYUC registry: development of a score for predicting MINOCA. *Vascular Health and Risk Management*.

[6] Iglesias-Garriz I, Delgado I, Prieto-Salvador I, Garrote C, García-Palomo A, Fernández-Vazquez F (2020). Previously diagnosed cancer and mortality after ST-segment elevation acute myocardial infarction treated with primary angioplasty. *Catheterization and Cardiovascular Interventions*.

[7] Donisan T, Balanescu DV, Palaskas N, Lopez-Mattei J, Karimzad K, Kim P (2019). Cardiac Interventional Procedures in Cardio-Oncology Patients. *Cardiology Clinics*.

[8] Giza DE, Lopez-Mattei J, Vejpongsa P, Munoz E, Iliescu G, Kitkungvan D (2017). Stress-Induced Cardiomyopathy in Cancer Patients. *The American Journal of Cardiology*.

[9] Giza DE, Boccalandro F, Lopez-Mattei J, Iliescu G, Karimzad K, Kim P (2017). Ischemic Heart Disease: Special Considerations in Cardio-Oncology. *Current Treatment Options in Cardiovascular Medicine*.

[10] Hayes SN, Kim ESH, Saw J, Adlam D, Arslanian-Engoren C, Economy KE (2018). Spontaneous Coronary Artery Dissection: Current State of the Science: a Scientific Statement from the American Heart Association. *Circulation*.

[11] Herrmann J, Yang EH, Iliescu CA, Cilingiroglu M, Charitakis K, Hakeem A (2016). Vascular Toxicities of Cancer Therapies: The Old and the New–An Evolving Avenue. *Circulation*.

[12] Tamis-Holland JE, Jneid H, Reynolds HR, Agewall S, Brilakis ES, Brown TM (2019). Contemporary Diagnosis and Management of Patients with Myocardial Infarction in the Absence of Obstructive Coronary Artery Disease: a Scientific Statement from the American Heart Association. *Circulation*.

[13] Thygesen K, Alpert JS, Jaffe AS, Chaitman BR, Bax JJ, Morrow DA (2018). Fourth Universal Definition of Myocardial Infarction (2018). *Journal of the American College of Cardiology*.

[14] Collet JP, Thiele H, Barbato E, Barthélémy O, Bauersachs J, Bhatt DL (2021). 2020 ESC Guidelines for the management of acute coronary syndromes in patients presenting without persistent ST-segment elevation: The Task Force for the management of acute coronary syndromes in patients presenting without persistent ST-segment elevation of the European Society of Cardiology (ESC). *European Heart Journal*.

[15] Agewall S, Eurenius L, Hofman-Bang C, Malmqvist K, Frick M, Jernberg T (2011). Myocardial infarction with angiographically normal coronary arteries. *Atherosclerosis*.

[16] Curzen N, Rana O, Nicholas Z, Golledge P, Zaman A, Oldroyd K (2014). Does Routine Pressure Wire Assessment Influence Management Strategy at Coronary Angiography for Diagnosis of Chest Pain?: the RIPCORD study. *Circulation: Cardiovascular Interventions*.

[17] Nappi AG, Boden WE (2016). Does Physiology Trump Anatomy as the “Best Course” to Guide PCI Decision Making and Outcomes. *Journal of the American College of Cardiology*.

[18] Zhang L, Awadalla M, Mahmood SS, Nohria A, Hassan MZO, Thuny F (2020). Cardiovascular magnetic resonance in immune checkpoint inhibitor-associated myocarditis. *European Heart Journal*.

[19] Pasupathy S, Air T, Dreyer RP, Tavella R, Beltrame JF (2015). Systematic Review of Patients Presenting with Suspected Myocardial Infarction and Nonobstructive Coronary Arteries. *Circulation*.

[20] Dawson DK (2018). Acute stress-induced (takotsubo) cardiomyopathy. *Heart*.

[21] Liu VY, Agha AM, Lopez-Mattei J, Palaskas N, Kim P, Thompson K (2018). Interventional Cardio-Oncology: Adding a New Dimension to the Cardio-Oncology Field. *Frontiers in Cardiovascular Medicine*.

[22] Munoz E, Iliescu G, Vejpongsa P, Charitakis K, Karimzad K, Lopez-Mattei J (2016). Takotsubo Stress Cardiomyopathy: ”Good News” in Cancer Patients. *Journal of the American College of Cardiology*.

[23] Bonaca MP, Olenchock BA, Salem J, Wiviott SD, Ederhy S, Cohen A (2019). Myocarditis in the Setting of Cancer Therapeutics: Proposed Case Definitions for Emerging Clinical Syndromes in Cardio-Oncology. *Circulation*.

[24] Whitlock MC, Yeboah J, Burke GL, Chen H, Klepin HD, Hundley WG (2015). Cancer and Its Association With the Development of Coronary Artery Calcification: An Assessment From the Multi-Ethnic Study of Atherosclerosis. *Journal of the American Heart Association*.

[25] Barac A, Murtagh G, Carver JR, Chen MH, Freeman AM, Herrmann J (2015). Cardiovascular Health of Patients with Cancer and Cancer Survivors: A Roadmap to the Next Level. *Journal of the American College of Cardiology*.

[26] Winther JF, Bhatia S, Cederkvist L, Gudmundsdottir T, Madanat-Harjuoja L, Tryggvadottir L (2018). Risk of cardiovascular disease among Nordic childhood cancer survivors with diabetes mellitus: a report from adult life after childhood cancer in Scandinavia. *Cancer*.

[27] Oren O, Herrmann J (2018). Arterial events in cancer patients—the case of acute coronary thrombosis. *Journal of Thoracic Disease*.

[28] Giza DE, Iliescu G, Hassan S, Marmagkiolis K, Iliescu C (2017). Cancer as a Risk Factor for Cardiovascular Disease. *Current Oncology Reports*.

[29] Banasiak W, Zymliński R, Undas A (2018). Optimal management of cancer patients with acute coronary syndrome. *Polish Archives of Internal Medicine*.

[30] Niccoli G, Scalone G, Crea F (2015). Acute myocardial infarction with no obstructive coronary atherosclerosis: mechanisms and management. *European Heart Journal*.

[31] Opolski MP, Spiewak M, Marczak M, Debski A, Knaapen P, Schumacher SP (2019). Mechanisms of Myocardial Infarction in Patients with Nonobstructive Coronary Artery Disease: Results From the Optical Coherence Tomography Study. *JACC: Cardiovascular Imaging*.

[32] Libby P, Pasterkamp G, Crea F, Jang I (2019). Reassessing the Mechanisms of Acute Coronary Syndromes. *Circulation Research*.

[33] Abdol Razak NB, Jones G, Bhandari M, Berndt MC, Metharom P (2018). Cancer-Associated Thrombosis: An Overview of Mechanisms, Risk Factors, and Treatment. *Cancers*.

[34] Navi BB, Reiner AS, Kamel H, Iadecola C, Okin PM, Elkind MSV (2017). Risk of Arterial Thromboembolism in Patients with Cancer. *Journal of the American College of Cardiology*.

[35] Connolly GC, Phipps RP, Francis CW (2014). Platelets and Cancer-Associated Thrombosis. *Seminars in Oncology*.

[36] van Es N, Sturk A, Middeldorp S, Nieuwland R (2014). Effects of Cancer on Platelets. *Seminars in Oncology*.

[37] Mezouar S, Frère C, Darbousset R, Mege D, Crescence L, Dignat-George F (2016). Role of platelets in cancer and cancer-associated thrombosis: Experimental and clinical evidences. *Thrombosis Research*.

[38] Demers M, Wagner DD (2014). NETosis: a new factor in tumor progression and cancer-associated thrombosis. *Seminars in Thrombosis and Hemostasis*.

[39] Thålin C, Demers M, Blomgren B, Wong SL, von Arbin M, von Heijne A (2016). NETosis promotes cancer-associated arterial microthrombosis presenting as ischemic stroke with troponin elevation. *Thrombosis Research*.

[40] Iliescu C, Balanescu DV, Donisan T, Giza DE, Muñoz Gonzalez ED, Cilingiroglu M (2018). Safety of Diagnostic and Therapeutic Cardiac Catheterization in Cancer Patients With Acute Coronary Syndrome and Chronic Thrombocytopenia. *The American Journal of Cardiology*.

[41] Aronson D, Brenner B (2018). Arterial thrombosis and cancer. *Thrombosis Research*.

[42] Baselet B, Rombouts C, Benotmane AM, Baatout S, Aerts A (2016). Cardiovascular diseases related to ionizing radiation: the risk of low-dose exposure (Review). *International Journal of Molecular Medicine*.

[43] Panella M, Ross JE, Garvin K, Martin A (2010). Cardiac Sudden Death as a Result of Acute Coronary Artery Thrombosis during Chemotherapy for Testicular Carcinoma. *Journal of Forensic Sciences*.

[44] Cameron AC, McMahon K, Hall M, Neves KB, Rios FJ, Montezano AC (2020). Comprehensive Characterization of the Vascular Effects of Cisplatin-Based Chemotherapy in Patients with Testicular Cancer. *JACC: CardioOncology*.

[45] Kobo OM, Vainer Evgrafov E, Cohen Y, Lerner Y, Khatib A, Hoffman R (2019). Non-ST-Elevation Myocardial Infarction with Non-significant Coronary Artery Disease as a Symptom of Occult or New Malignancy. *The Israel Medical Association Journal*.

[46] Balanescu DV, Monlezun DJ, Donisan T, Boone D, Cervoni-Curet F, Palaskas N (2019). A Cancer Paradox: Machine-Learning Backed Propensity-Score Analysis of Coronary Angiography Findings in Cardio-Oncology. *The Journal of Invasive Cardiology*.

[47] Cautela J, Rouby F, Salem J, Alexandre J, Scemama U, Dolladille C (2020). Acute Coronary Syndrome with Immune Checkpoint Inhibitors: a Proof-of-Concept Case and Pharmacovigilance Analysis of a Life-Threatening Adverse Event. *Canadian Journal of Cardiology*.

[48] Tomita Y, Sueta D, Kakiuchi Y, Saeki S, Saruwatari K, Sakata S (2017). Acute coronary syndrome as a possible immune-related adverse event in a lung cancer patient achieving a complete response to anti-PD-1 immune checkpoint antibody. *Annals of Oncology*.

[49] Masson R, Manthripragada G, Liu R, Tavakoli J, Mok K (2020). Possible Precipitation of Acute Coronary Syndrome with Immune Checkpoint Blockade: A Case Report. *The Permanente Journal*.

[50] Bar J, Markel G, Gottfried T, Percik R, Leibowitz-Amit R, Berger R (2019). Acute vascular events as a possibly related adverse event of immunotherapy: a single-institute retrospective study. *European Journal of Cancer*.

[51] Solinas C, Saba L, Sganzerla P, Petrelli F (2020). Venous and arterial thromboembolic events with immune checkpoint inhibitors: a systematic review. *Thrombosis Research*.

[52] Newman JL, Stone JR (2019). Immune checkpoint inhibition alters the inflammatory cell composition of human coronary artery atherosclerosis. *Cardiovascular Pathology*.

[53] Jaiswal S, Natarajan P, Silver AJ, Gibson CJ, Bick AG, Shvartz E (2017). Clonal Hematopoiesis and Risk of Atherosclerotic Cardiovascular Disease. *New England Journal of Medicine*.

[54] Sperling AS, Gibson CJ, Ebert BL (2017). The genetics of myelodysplastic syndrome: from clonal haematopoiesis to secondary leukaemia. *Nature Reviews Cancer*.

[55] Montone RA, Niccoli G, Fracassi F, Russo M, Gurgoglione F, Cammà G (2018). Patients with acute myocardial infarction and non-obstructive coronary arteries: safety and prognostic relevance of invasive coronary provocative tests. *European Heart Journal*.

[56] Shepherd JT, Vanhoutte PM (1985). Spasm of the Coronary Arteries: Causes and Consequences (the Scientist’s Viewpoint). *Mayo Clinic Proceedings*.

[57] Tsibiribi P, Bui-Xuan C, Bui-Xuan B, Lombard-Bohas C, Duperret S, Belkhiria M (2006). Cardiac lesions induced by 5-fluorouracil in the rabbit. *Human and Experimental Toxicology*.

[58] Kumar S, Gupta RK, Samal N (1995). 5-fluorouracil induced cardiotoxicity in albino rats. *Polish Journal of Medicine and Pharmacy*.

[59] Meyer CC, Calis KA, Burke LB, Walawander CA, Grasela TH (1997). Symptomatic cardiotoxicity associated with 5-fluorouracil. *Pharmacotherapy*.

[60] Südhoff T, Enderle M-, Pahlke M, Petz C, Teschendorf C, Graeven U (2004). 5-Fluorouracil induces arterial vasocontractions. *Annals of Oncology*.

[61] Jensen SA, Hasbak P, Mortensen J, Sørensen JB (2010). Fluorouracil Induces Myocardial Ischemia with Increases of Plasma Brain Natriuretic Peptide and Lactic Acid but without Dysfunction of Left Ventricle. *Journal of Clinical Oncology*.

[62] Saif MW, Shah MM, Shah AR (2009). Fluoropyrimidine-associated cardiotoxicity: revisited. *Expert Opinion on Drug Safety*.

[63] Meydan N, Kundak I, Yavuzsen T, Oztop I, Barutca S, Yilmaz U (2005). Cardiotoxicity of de Gramont’s regimen: incidence, clinical characteristics and long-term follow-up. *Japanese Journal of Clinical Oncology*.

[64] Polk A, Vaage-Nilsen M, Vistisen K, Nielsen DL (2013). Cardiotoxicity in cancer patients treated with 5-fluorouracil or capecitabine: a systematic review of incidence, manifestations and predisposing factors. *Cancer Treatment Reviews*.

[65] Wacker A, Lersch C, Scherpinski U, Reindl L, Seyfarth M (2003). High Incidence of Angina pectoris in Patients Treated with 5-Fluorouracil. A planned surveillance study with 102 patients. *Oncology*.

[66] Shoemaker LK, Arora U, Rocha Lima CMS (2004). 5-Fluorouracil-Induced Coronary Vasospasm. *Cancer Control*.

[67] Yuan C, Parekh H, Allegra C, George TJ, Starr JS (2019). 5-FU induced cardiotoxicity: case series and review of the literature. *Cardio-Oncology*.

[68] Kim S, Kwak C, Lee B, Kim SB, Sir J, Cho W (2012). A Case of Severe Coronary Spasm Associated with 5-Fluorouracil Chemotherapy. *The Korean Journal of Internal Medicine*.

[69] Tsiamis E, Synetos A, Stefanadis C (2012). Capecitabine may induce coronary artery vasospasm. *Hellenic Journal of Cardiology*.

[70] Tajik R, Saadat H, Taherkhani M, Movahed MR (2010). Angina Induced by 5-Fluorouracil Infusion in a Patient with Normal Coronaries. *The American Heart Hospital Journal*.

[71] Alter P, Herzum M, Soufi M, Schaefer J, Maisch B (2006). Cardiotoxicity of 5-Fluorouracil. *Cardiovascular and Hematological Agents in Medicinal Chemistry*.

[72] Camaro C, Danse PW, Bosker HA (2009). Acute chest pain in a patient treated with capecitabine. *Netherlands Heart Journal*.

[73] Sara JD, Kaur J, Khodadadi R, Rehman M, Lobo R, Chakrabarti S (2018). 5-fluorouracil and cardiotoxicity: a review. *Therapeutic Advances in Medical Oncology*.

[74] Dalzell JR, Samuel LM (2009). The spectrum of 5-fluorouracil cardiotoxicity. *Anti-Cancer Drugs*.

[75] Luwaert RJ, Descamps O, Majois F, Chaudron J, Beauduin M (1991). Coronary artery spasm induced by 5-fluorouracil. *European Heart Journal*.

[76] Ghosh AK, Crake T, Manisty C, Westwood M (2018). Pericardial Disease in Cancer Patients. *Current Treatment Options in Cardiovascular Medicine*.

[77] de Forni M, Malet-Martino MC, Jaillais P, Shubinski RE, Bachaud JM, Lemaire L (1992). Cardiotoxicity of high-dose continuous infusion fluorouracil: a prospective clinical study. *Journal of Clinical Oncology*.

[78] Saneeymehri SS, Markey KR, Mahipal A (2016). Paradoxical effect of capecitabine in 5-fluorouracil-induced cardiotoxicity: a case vignette and literature review. *Journal of Oncology Pharmacy Practice*.

[79] Clasen SC, Ky BX, O’Quinn R, Giantonio B, Teitelbaum U, Carver JR (2017). Fluoropyrimidine-induced cardiac toxicity: challenging the current paradigm. *Journal of Gastrointestinal Oncology*.

[80] Kosmas C, Kallistratos MS, Kopterides P, Syrios J, Skopelitis H, Mylonakis N (2008). Cardiotoxicity of fluoropyrimidines in different schedules of administration: a prospective study. *Journal of Cancer Research and Clinical Oncology*.

[81] Akpek G, Hartshorn KL (1999). Failure of oral nitrate and calcium channel blocker therapy to prevent 5-fluorouracil-related myocardial ischemia: a case report. *Cancer Chemotherapy and Pharmacology*.

[82] Sasaki W, Wada H, Sakakura K, Matsuda J, Ibe T, Hayashi T (2020). Coronary vasospasm induced by cisplatin for seminoma. *Clinical Case Reports*.

[83] Hanchate LP, Sharma SR, Madyalkar S (2017). Cisplatin Induced Acute Myocardial Infarction and Dyslipidemia. *Journal of Clinical and Diagnostic Research*.

[84] Rao A, Kumar R, Narayanan G (2015). A rare case of cisplatin-induced acute myocardial infarction in a patient receiving chemoradiation for lung cancer. *Journal of Cancer Research and Therapeutics*.

[85] Fukuda M, Oka M, Itoh N, Sakamoto T, Mori H, Hayakawa A (1999). Vasospastic Angina Likely Related to Cisplatin-containing Chemotherapy and Thoracic Irradiation for Lung Cancer. *Internal Medicine*.

[86] Stefenelli T, Kuzmits R, Ulrich W, Glogar D (1988). Acute vascular toxicity after combination chemotherapy with cisplatin, vinblastine, and bleomycin for testicular cancer. *European Heart Journal*.

[87] Hassan SA, Palaskas N, Kim P, Iliescu C, Lopez-Mattei J, Mouhayar E (2018). Chemotherapeutic Agents and the Risk of Ischemia and Arterial Thrombosis. *Current Atherosclerosis Reports*.

[88] Gupta S, Ghosh J, Bajpai J, Maheshwari A, Shah K (2012). Acute non-ST elevation myocardial infarction following paclitaxel administration for ovarian carcinoma: a case report and review of literature. *Journal of Cancer Research and Therapeutics*.

[89] Gemici G, Çinçin A, Deǧertekin M, Oktay A (2009). Paclitaxel-induced ST-Segment Elevations. *Clinical Cardiology*.

[90] Rawal G, Yadav S, Kumar R (2016). Paclitaxel Induced Acute ST Elevation Myocardial Infarction: A Rare Case Report. *Journal of Clinical and Diagnostic Research*.

[91] McGuire WP (1989). Taxol: a Unique Antineoplastic Agent with Significant Activity in Advanced Ovarian Epithelial Neoplasms. *Annals of Internal Medicine*.

[92] Wada H, Suzuki M, Matsuda M, Ajiro Y, Shinozaki T, Sakagami S (2018). VEGF-C and Mortality in Patients With Suspected or Known Coronary Artery Disease. *Journal of the American Heart Association*.

[93] Huang A, Qi X, Cui Y, Wu Y, Zhou S, Zhang M (2020). Serum VEGF: Diagnostic Value of Acute Coronary Syndrome from Stable Angina Pectoris and Prognostic Value of Coronary Artery Disease. *Cardiology Research and Practice*.

[94] Sudasena D, Balanescu DV, Donisan T, Hassan S, Palaskas N, Kim P (2019). Fulminant Vascular and Cardiac Toxicity Associated with Tyrosine Kinase Inhibitor Sorafenib. *Cardiovascular Toxicology*.

[95] Fiets RB, Staal AHJ, Cramer GE, Blijlevens NMA (2018). Coronary artery spasms due to tyrosine kinase inhibitors used in chronic myeloid leukemia. *The Netherlands Journal of Medicine*.

[96] Moslehi JJ, Deininger M (2015). Tyrosine Kinase Inhibitor-Associated Cardiovascular Toxicity in Chronic Myeloid Leukemia. *Journal of Clinical Oncology*.

[97] Touyz RM, Herrmann J (2018). Cardiotoxicity with vascular endothelial growth factor inhibitor therapy. *NPJ Precision Oncology*.

[98] Chen-Scarabelli C, Corsetti G, Pasini E, Dioguardi FS, Sahni G, Narula J (2017). Spasmogenic Effects of the Proteasome Inhibitor Carfilzomib on Coronary Resistance, Vascular Tone and Reactivity. *EBioMedicine*.

[99] Takamatsu H, Yamashita T, Kotani T, Sawazaki A, Okumura H, Nakao S (2010). Ischemic heart disease associated with bortezomib treatment combined with dexamethasone in a patient with multiple myeloma. *International Journal of Hematology*.

[100] Yasui T, Shioyama W, Oboshi M, Nishikawa T, Kamada R, Oka T (2020). Coronary spastic angina in a multiple myeloma patient treated with bortezomib, lenalidomide, and dexamethasone. *Journal of Cardiology Cases*.

[101] Miller DD, Waters DD, Dangoisse V, David P (1983). Symptomatic Coronary Artery Spasm Following Radiotherapy for Hodgkin’s Disease. *Chest*.

[102] Yahalom J, Hasin Y, Fuks Z (1983). Acute myocardial infarction with normal coronary arteriogram after mantle field radiation therapy for Hodgkin’s disease. *Cancer*.

[103] Herrmann J (2020). Vascular toxic effects of cancer therapies. *Nature Reviews Cardiology*.

[104] Soloviev AI, Tishkin SM, Parshikov AV, Ivanova IV, Goncharov EV, Gurney AM (2003). Mechanisms of endothelial dysfunction after ionized radiation: selective impairment of the nitric oxide component of endothelium-dependent vasodilation. *British Journal of Pharmacology*.

[105] Sugihara T, Hattori Y, Yamamoto Y, Qi F, Ichikawa R, Sato A (1999). Preferential Impairment of Nitric Oxide-Mediated Endothelium-Dependent Relaxation in Human Cervical Arteries after Irradiation. *Circulation*.

[106] Levesque L, Lam M, Allaire P, Mondat M, Houle S, Beaudoin G (2001). Effects of Radiation Therapy on Vascular Responsiveness. *Journal of Cardiovascular Pharmacology*.

[107] Beltrame JF, Crea F, Camici P (2009). Advances in Coronary Microvascular Dysfunction. *Heart, Lung and Circulation*.

[108] Ong P, Camici PG, Beltrame JF, Crea F, Shimokawa H, Sechtem U (2018). International standardization of diagnostic criteria for microvascular angina. *International Journal of Cardiology*.

[109] Toya T, Sara JD, Corban MT, Taher R, Godo S, Herrmann J (2020). Assessment of peripheral endothelial function predicts future risk of solid-tumor cancer. *European Journal of Preventive Cardiology*.

[110] Herrmann J, Kaski JC, Lerman A (2012). Coronary microvascular dysfunction in the clinical setting: from mystery to reality. *European Heart Journal*.

[111] Economopoulou P, Kotsakis A, Kapiris I, Kentepozidis N (2015). Cancer therapy and cardiovascular risk: focus on bevacizumab. *Cancer Management and Research*.

[112] Sane DC, Anton L, Brosnihan KB (2004). Angiogenic growth factors and hypertension. *Angiogenesis*.

[113] Kamba T, McDonald DM (2007). Mechanisms of adverse effects of anti-VEGF therapy for cancer. *British Journal of Cancer*.

[114] Totzeck M, Mincu RI, Rassaf T (2017). Cardiovascular Adverse Events in Patients with Cancer Treated with Bevacizumab: a Meta-Analysis of more than 20 000 Patients. *Journal of the American Heart Association*.

[115] Chen C, Yamaguchi H, Lee H, Du Y, Lee H, Xia W (2011). Dual Targeting of Tumor Angiogenesis and Chemotherapy by Endostatin-Cytosine Deaminase-Uracil Phosphoribosyltransferase. *Molecular Cancer Therapeutics*.

[116] Bordun K, Premecz S, daSilva M, Mandal S, Goyal V, Glavinovic T (2015). The utility of cardiac biomarkers and echocardiography for the early detection of bevacizumab- and sunitinib-mediated cardiotoxicity. *American Journal of Physiology-Heart and Circulatory Physiology*.

[117] Oladiran O, Nazir S (2018). Bevacizumab: a Rare Cause of Nonischemic Cardiomyopathy. *Case Reports in Cardiology*.

[118] Katoh M, Takeda N, Arimoto T, Abe H, Oda K, Osuga Y (2017). Bevacizumab-Related Microvascular Angina and its Management with Nicorandil. *International Heart Journal*.

[119] Chintalgattu V, Rees ML, Culver JC, Goel A, Jiffar T, Zhang J (2013). Coronary Microvascular Pericytes are the Cellular Target of Sunitinib Malate–Induced Cardiotoxicity. *Science Translational Medicine*.

[120] Latifi Y, Moccetti F, Wu M, Xie A, Packwood W, Qi Y (2019). Thrombotic microangiopathy as a cause of cardiovascular toxicity from the BCR-ABL1 tyrosine kinase inhibitor ponatinib. *Blood*.

[121] Wu MD, Hodovan J, Kumar K, Moulton B, Olson S, Gilbert A (2020). Ponatinib coronary microangiopathy: novel bedside diagnostic approach and management with N-acetylcysteine. *Blood Advances*.

[122] Andersen R, Wethal T, Günther A, Fosså A, Edvardsen T, Fosså SD (2010). Relation of coronary artery calcium score to premature coronary artery disease in survivors >15 years of Hodgkin’s lymphoma. *The American Journal of Cardiology*.

[123] Fajardo LF (1989). The unique physiology of endothelial cells and its implications in radiobiology. *Frontiers of Radiation Therapy and Oncology*.

[124] Walaszczyk A, Szołtysek K, Jelonek K, Polańska J, Dörr W, Haagen J (2018). Heart irradiation reduces microvascular density and accumulation of HSPA1 in mice. *Strahlentherapie Und Onkologie*.

[125] Amromin GD, Gildenhorn HL, Solomon RD, Nadkarni BB, Jacobs ML (1964). The synergism of x-irradiation and cholesterol-fat feeding on the development of coronary artery lesions. *Journal of Atherosclerosis Research*.

[126] Darby SC, Ewertz M, McGale P, Bennet AM, Blom-Goldman U, Brønnum D (2013). Risk of ischemic heart disease in women after radiotherapy for breast cancer. *The New England Journal of Medicine*.

[127] Seemann I, te Poele JAM, Song J, Hoving S, Russell NS, Stewart FA (2013). Radiation- and anthracycline-induced cardiac toxicity and the influence of ErbB2 blocking agents. *Breast Cancer Research and Treatment*.

[128] Hader SN, Zinkevich N, Norwood Toro LE, Kriegel AJ, Kong A, Freed JK (2019). Detrimental effects of chemotherapy on human coronary microvascular function. *American Journal of Physiology-Heart and Circulatory Physiology*.

[129] Sandoval Y, Smith SW, Thordsen SE, Apple FS (2014). Supply/Demand Type 2 Myocardial Infarction: should we be paying more attention. *Journal of the American College of Cardiology*.

[130] Nestelberger T, Boeddinghaus J, Badertscher P, Twerenbold R, Wildi K, Breitenbücher D (2017). Effect of Definition on Incidence and Prognosis of Type 2 Myocardial Infarction. *Journal of the American College of Cardiology*.

[131] Balanescu DV, Donisan T, Deswal A, Palaskas N, Song J, Lopez-Mattei J (2020). Acute myocardial infarction in a high-risk cancer population: Outcomes following conservative versus invasive management. *International Journal of Cardiology*.

[132] Vora AN, Wang TY, Hellkamp AS, Thomas L, Henry TD, Goyal A (2016). Differences in Short- and Long-Term Outcomes among Older Patients with ST-Elevation Versus Non-ST-Elevation Myocardial Infarction with Angiographically Proven Coronary Artery Disease. *Circulation: Cardiovascular Quality and Outcomes*.

[133] Park JY, Guo W, Al-Hijji M, El Sabbagh A, Begna KH, Habermann TM (2019). Acute coronary syndromes in patients with active hematologic malignancies - Incidence, management, and outcomes. *International Journal of Cardiology*.

[134] Saw J, Mancini GBJ, Humphries KH (2016). Contemporary Review on Spontaneous Coronary Artery Dissection. *Journal of the American College of Cardiology*.

[135] Hayes SN, Tweet MS, Adlam D, Kim ESH, Gulati R, Price JE (2020). Spontaneous Coronary Artery Dissection: JACC State-of-the-Art Review. *Journal of the American College of Cardiology*.

[136] Waterbury TM, Tweet MS, Hayes SN, Eleid MF, Bell MR, Lerman A (2018). Early Natural History of Spontaneous Coronary Artery Dissection. *Circulation: Cardiovascular Interventions*.

[137] Waterbury TM, Tarantini G, Vogel B, Mehran R, Gersh BJ, Gulati R (2020). Non-atherosclerotic causes of acute coronary syndromes. *Nature Reviews Cardiology*.

[138] Choi S, Nam C, Bae H, Cho Y, Yoon H, Hur S (2013). Spontaneous coronary artery dissection diagnosed by intravascular ultrasound and followed up by cardiac computed tomography. *The Korean Journal of Internal Medicine*.

[139] Saw J, Aymong E, Sedlak T, Buller CE, Starovoytov A, Ricci D (2014). Spontaneous Coronary Artery Dissection: association with predisposing arteriopathies and precipitating stressors and cardiovascular outcomes. *Circulation: Cardiovascular Interventions*.

[140] Krittanawong C, Kumar A, Johnson KW, Luo Y, Yue B, Wang Z (2019). Conditions and Factors Associated with Spontaneous Coronary Artery Dissection (from a National Population-Based Cohort Study). *The American Journal of Cardiology*.

[141] Tweet MS, Hayes SN, Pitta SR, Simari RD, Lerman A, Lennon RJ (2012). Clinical Features, Management, and Prognosis of Spontaneous Coronary Artery Dissection. *Circulation*.

[142] Abbott JD, Curtis JP, Murad K, Kramer HM, Remetz MS, Setaro JF (2003). Spontaneous Coronary Artery Dissection in a Woman Receiving 5-Fluorouracil–a case report. *Angiology*.

[143] Hart K, Patel S, Kovoor J (2019). Spontaneous Coronary Artery Dissection Associated with Anal Cancer Management with Fluorouracil and Radiotherapy. *Cureus*.

[144] Ghosh N, Chow C, Korley V, Chisholm R (2008). An unusual case of chronic coronary artery dissection: did cisplatin play a role. *Canadian Journal of Cardiology*.

[145] Mir MA, Patnaik MM, Herrmann J (2013). Spontaneous coronary artery dissection during hematopoietic stem cell infusion. *Blood*.

[146] Karabay CY, Biteker M, Zehir R, Bezgin T, Tanboga H, Can MM (2010). Multiple spontaneous coronary artery dissection associated with Trousseau’s syndrome. *Cardiology Journal*.

[147] Kok SN, Hayes SN, Cutrer FM, Raphael CE, Gulati R, Best PJM (2018). Prevalence and Clinical Factors of Migraine in Patients with Spontaneous Coronary Artery Dissection. *Journal of the American Heart Association*.

[148] Saw J, Starovoytov A, Humphries K, Sheth T, So D, Minhas K (2019). Canadian spontaneous coronary artery dissection cohort study: in-hospital and 30-day outcomes. *European Heart Journal*.

[149] Daniels K, Abma J (2018). Current contraceptive status among women aged 15–49: United States, 2015–2017. *NCHS Data Brief*.

[150] Sprague BL, Trentham-Dietz A, Cronin KA (2012). A Sustained Decline in Postmenopausal Hormone Use: results from the National Health and Nutrition Examination Survey, 1999–2010. *Obstetrics and Gynecology*.

[151] Weinberg BA, Pinkerton CA, Waller BF (1990). External compression by metastatic squamous cell carcinoma: a rare cause of left main coronary artery narrowing. *Clinical Cardiology*.

[152] Rehman KA, Betancor J, Xu B, Tan CD, Rodriguez ER, Asher CR (2017). An Unusual Cause of Acute Myocardial Infarction Caused by a Large Pulmonary Artery Intimal Sarcoma. *CASE*.

[153] Gue YX, Anwar M, Gorog DA (2018). A rare cause of myocardial infarction with non-obstructive coronary arteries-case report of ST-segment elevation myocardial infarction caused by a mediastinal mass. *European Heart Journal - Case Reports*.

[154] Lang NN, Newby DE (2012). Cardiac compression causing fatal acute ST-segment elevation myocardial infarction. *QJM*.

[155] Tornvall P, Gerbaud E, Behaghel A, Chopard R, Collste O, Laraudogoitia E (2015). Myocarditis or “true” infarction by cardiac magnetic resonance in patients with a clinical diagnosis of myocardial infarction without obstructive coronary disease: a meta-analysis of individual patient data. *Atherosclerosis*.

[156] Desai A, Noor A, Joshi S, Kim AS (2019). Takotsubo cardiomyopathy in cancer patients. *Cardio-Oncology*.

[157] Madias JE (2016). What is/are the trigger(s) of takotsubo syndrome in cancer patients receiving chemotherapy. *International Journal of Cardiology*.

[158] Ederhy S, Dolladille C, Thuny F, Alexandre J, Cohen A (2019). Takotsubo syndrome in patients with cancer treated with immune checkpoint inhibitors: a new adverse cardiac complication. *European Journal of Heart Failure*.

[159] Tse YH, Chan WS, Chim CS, Tse HF (2021). Lenalidomide-induced focal myocarditis mimicking acute ST segment elevation myocardial infarction. *Postgraduate Medical Journal*.

[160] Jacob R, Strati P, Palaskas N, Lopez-Mattei JC, Marmagkiolis K, Buja LM (2020). Lenalidomide-Induced Myocarditis, Rare But Possibly Fatal Toxicity of a Commonly Used Immunotherapy. *JACC: Case Reports*.

[161] Fazio G, Pizzuto C, Barbaro G, Sutera L, Incalcaterra E, Evola G (2008). Chronic pharmacological treatment in takotsubo cardiomyopathy. *International Journal of Cardiology*.

[162] Ewer MS, Lippman SM (2005). Type II chemotherapy-related cardiac dysfunction: time to recognize a new entity. *Journal of Clinical Oncology*.

